# 

**Published:** 1885-07

**Authors:** 


					﻿Vhole Numbej, ^07.
JULY, 1885.
Vo1
TZETZZE
CHICAGO MEDICAL JOURNAL AND EXAMINER
EDITORS:
S. J. JONES. M D LLD
W. W. JAGGARD, A.M , M.D.
HAROLD N. MOYER, M.D
CHICAGO
PUBLISHED MONTHLY BY THE CHICAGO MEDICAL PRESS ASSOCIATION
240 Wabash Avenue.
CLARK & LONGLEY, PRINTERS, 63 AND 165 DEARBORN STREET, CHICAGO.
				

